# Further Insights into the Gut Microbiota of Cow’s Milk Allergic Infants: Analysis of Microbial Functionality and Its Correlation with Three Fecal Biomarkers

**DOI:** 10.3390/ijms24119247

**Published:** 2023-05-25

**Authors:** Elisa Zubeldia-Varela, Tomás Clive Barker-Tejeda, Leticia Mera-Berriatua, Raphaëlle Bazire, Paula Cabrera-Freitag, Carles Ubeda, Domingo Barber, María Pilar Francino, David Rojo, María Dolores Ibáñez-Sandín, Marina Pérez-Gordo

**Affiliations:** 1 Departamento de Ciencias Médicas Básicas, Instituto de Medicina Molecular Aplicada (IMMA) Nemesio Díez, Facultad de Medicina, Universidad San Pablo-CEU, CEU Universities, 28668 Boadilla del Monte, Spain; elisa.zubeldiavarela@ceu.es (E.Z.-V.);; 2 Centro de Metabolómica y Bioanálisis (CEMBIO), Facultad de Farmacia, Universidad San Pablo-CEU, CEU Universities, 28668 Boadilla del Monte, Spain; 3 Department of Allergy, H. Infantil Universitario Niño Jesús, FibHNJ, ARADyAL-RETICs Instituto de Salud Carlos III, IIS-P, 28031 Madrid, Spain; 4 Allergy Paediatric Unit, Allergy Service, Hospital General Universitario Gregorio Marañón, Gregorio Marañón Health Research Institute (IiSGM), 28007 Madrid, Spain; 5 Fundació per al Foment de la Investigació Sanitària i Biomèdica de la Comunitat Valenciana (FISABIO), 46020 Valencia, Spain; 6 CIBER en Epidemiología y Salud Pública, 28029 Madrid, Spain; 7 Joint Research Unit in Genomics and Health, Fundació per al Foment de la Investigació Sanitària i Biomèdica de la Comunitat Valenciana (FISABIO) and Institut de Biologia Integrativa de Sistemes (Universitat de València/Consejo Superior de Investigaciones Científicas), Avda. Catalunya 21, 46020 València, Spain

**Keywords:** gut microbiota, food allergy, cow’s milk allergy, shotgun metagenomics sequencing, fecal biomarkers, calprotectin, lactoferrin

## Abstract

Cow’s milk allergy (CMA) is one of the most prevalent food allergies in children. Several studies have demonstrated that gut microbiota influences the acquisition of oral tolerance to food antigens at initial stages of life. Changes in the gut microbiota composition and/or functionality (i.e., dysbiosis) have been linked to inadequate immune system regulation and the emergence of pathologies. Moreover, omic sciences have become an essential tool for the analysis of the gut microbiota. On the other hand, the use of fecal biomarkers for the diagnosis of CMA has recently been reviewed, with fecal calprotectin, α-1 antitrypsin, and lactoferrin being the most relevant. This study aimed at evaluating functional changes in the gut microbiota in the feces of cow’s milk allergic infants (AI) compared to control infants (CI) by metagenomic shotgun sequencing and at correlating these findings with the levels of fecal biomarkers (α-1 antitrypsin, lactoferrin, and calprotectin) by an integrative approach. We have observed differences between AI and CI groups in terms of fecal protein levels and metagenomic analysis. Our findings suggest that AI have altered glycerophospholipid metabolism as well as higher levels of lactoferrin and calprotectin that could be explained by their allergic status.

## 1. Introduction

The human microbiota is a complex ecosystem in which bacteria, fungi, viruses, archaea, and parasites cohabit within the human body. Among all its components, bacteria are the most abundant [1]. Among the body’s microbial ecosystems, the most complex, diverse, and numerous is the one associated with the gastrointestinal tract (GIT), where the density of microorganisms increases from the stomach to the colon [2]. Feces are representative of the microbiota composition of the colon, which, together with their noninvasive collection method, make them the biological sample of election for the study of the gut microbiota [3,4,5].

The gut microbiota composition changes throughout life. It is thought that 70% of the gut microbiota primary colonization occurs through maternal transmission [6,7], and that the first 1000 days of life, where the body encounters for the first time with external factors, are crucial [8]. Moreover, the development of the gut microbiota in the first years of life correlates with the development and maturation of the intestine and the immune system. From birth, there is a symbiotic relationship between the microbiota and human cells, which evolves over time, adapting to changes. After the first 2–3 years of life, the gut microbiota becomes similar to what it will be for the rest of the human’s life [1,9]. Notably, multiple factors such as age, nutrition, use of antibiotics, lifestyle, and environmental conditions have an impact on the gut microbiota’s dynamic composition [4,10,11].

The greatest source of stimulation of the immune system is found in the mucous surfaces of the body. About 70–80% of the immune system cells are found in the small and large intestines [12,13]. The gut microbiota stimulates and modulates the immune system by a dendritic cell-mediated immune regulation. Microbes promote the differentiation of regulatory T cells by the activation of dendritic cells present in the mucous surface of the intestine through the Toll-like receptor (TLR) pathway [14]. These activated cells produce cytokines that in turn activate naïve T cells or Th0 cells so that they mature towards the corresponding T cell subtype, Th1, Th2, Th17, or regulatory T cells. In healthy individuals, all Th cell subpopulations are present in a dynamic balance with regulatory T cells [15].

The prevalence of allergic diseases has increased in the last decades [16], with cow’s milk allergy (CMA) being one of the most prevalent food allergies in children. Several studies have demonstrated that the presence of certain bacteria in our GIT mediates the initiation of tolerogenic responses to antigens [17,18,19]. Dysbiosis—changes in the microbiota’s composition and/or functionality—has been linked to inadequate immune system regulation and the emergence of pathologies that are not just GIT-related. Several studies have shown a link between lung microbiota and respiratory allergy, as well as skin microbiota and the development of atopic dermatitis, and gut microbiota and food allergies [15,20,21,22,23].

It is not clear, however, whether the imbalance of the gut microbiota is a cause that triggers the disease or, on the contrary, it is a consequence of the disease that alters the bacterial populations and their functionality. To shed light on this issue, the omics technique of metagenomics has been used. Shotgun metagenomic sequencing offers an excellent approach to the study of the gut microbiota, as it allows researchers to determine its associated biological functions through DNA analysis [24,25]. This omic technique provides data about which metabolic pathways are encoded in the microbial genome, thus providing insight into microbial biological functions [26].

On the other hand, the use of fecal biomarkers for the diagnosis of CMA has recently been reviewed [27]. The proteins most studied as potential fecal biomarkers of this disease are fecal calprotectin, α-1 antitrypsin, and lactoferrin [28,29,30,31,32]. α1-Antitrypsin is a non-dietary serum protein synthesized in the liver [33]. It is resistant to digestive degradation and can therefore be reliably used to assess excessive gastrointestinal protein losses [32,34]. Lactoferrin is a globular protein belonging to the family of transferrin, non-heme iron-binding glycoproteins [35]. Lactoferrin was first described in mammalian milk [36]. It is also part of other secretions in the body and is produced by hematopoietic tissue in the bone marrow as part of neutrophil granules [37,38]. In addition, lactoferrin acts as a prominent component of the host’s first line of defense against infection and inflammation [39,40,41]. Finally, calprotectin is an immunomodulatory, antimicrobial, and antiproliferative protein that is present in the cytoplasm of neutrophils, in macrophage membranes, in activated monocytes, and in mucosal epithelial cells [42,43]. Among them, calprotectin has been the most discussed, and has been the subject of a recent systematic review for its use as a biomarker in CMA [44].

By using shotgun metagenomic sequencing, this study aimed to evaluate functional changes in the fecal gut microbiota of cow’s milk allergic infants (AI) compared to control infants (CI), aged between 4 and 6 months, and to determine the relationship between these findings and the levels of the fecal biomarkers α-1 antitrypsin, lactoferrin, and calprotectin.

## 2. Results

### 2.1. Gut Microbiota Functionality Profile Associated with CMA

**Figure 1:**
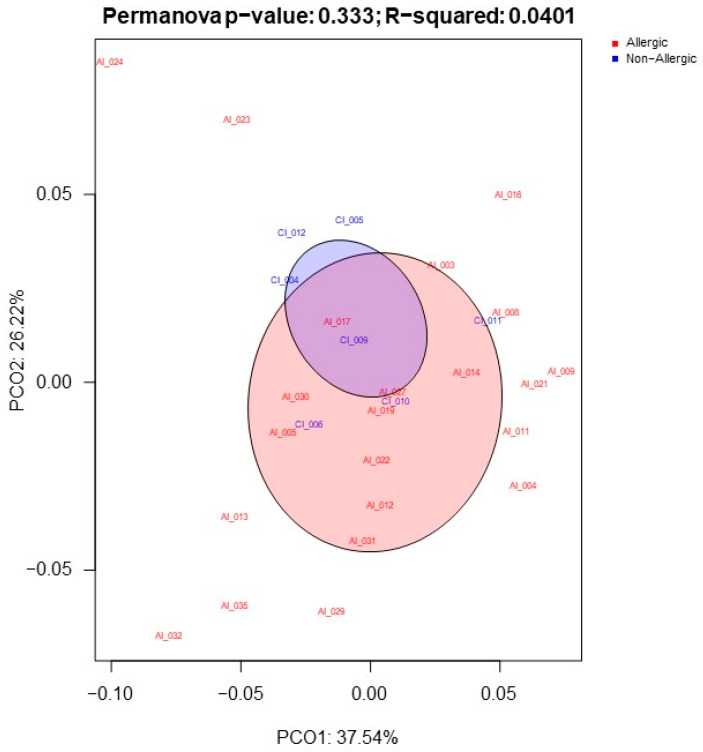
PCoA of Bray–Curtis dissimilarities at the metabolic pathway level. The analysis is based on the KEGG functional classification. Ellipses represent the experimental groups (AI and CI) and the clustering of the samples (Permanova *p*-value = 0.33). AI: allergic infants; CI: control infants.

For an overview of the link between gut microbiota functionality and the development of CMA, a principal coordinate analysis (PCoA) was performed between AI and CI groups (Figure 1), aiming at seeking associations between two sets of variables. These analyses did not show significant differences in the gut microbiome of AI and CI.

Results showed 313 metabolic pathways. Among them, 14 metabolic pathways were significantly different in relative abundance (*p*-value < 0.05) between AI and CI. All significant metabolic pathways with their corresponding statistical values are shown in Table 1.

**Figure 2:**
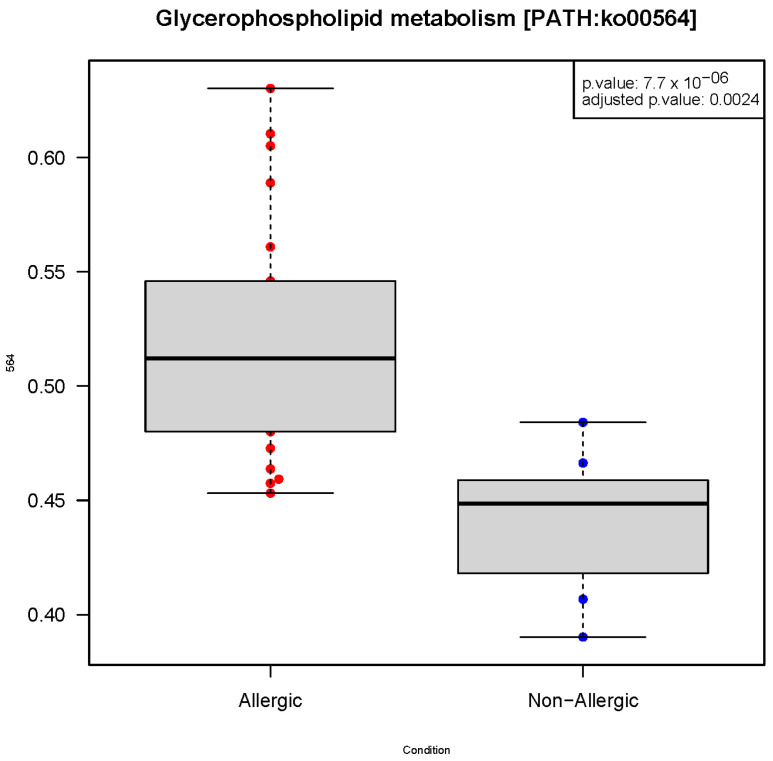
Box plot of the two groups of infants in relation to the abundance of glycerophospholipid metabolism counts. The *y*-axis shows the relative frequencies of the metabolic pathway reads. *p*-value = 7.7 × 10^−6^. The means and standard deviations of the groups were: Allergic = 0.521 ± 0.052 and Non-Allergic = 0.439 ± 0.033.

The only metabolic pathway with a significant adjusted *p*-value (<0.05) was glycerophospholipid metabolism. This metabolism was found to be increased in AI, as shown in the box plot in Figure 2.

### 2.2. Fecal Protein Immunodetection

**Figure 3:**
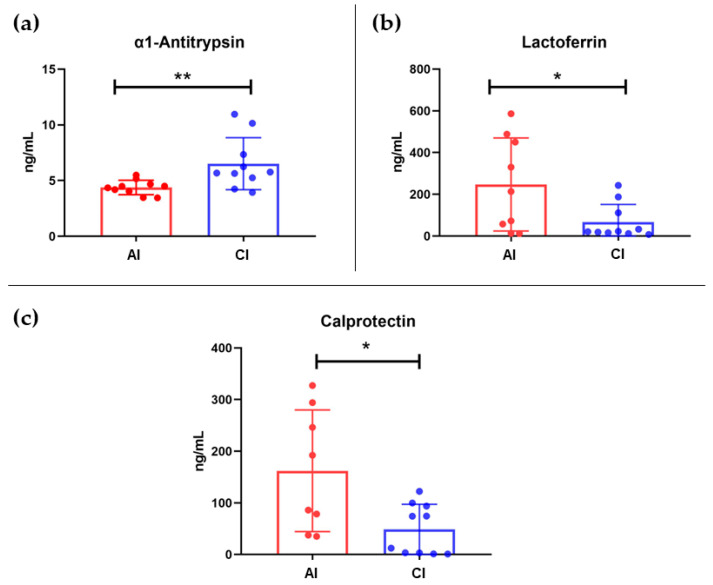
(**a**) Bar graph of α1-Antitrypsin ** *p*-value < 0.01 by Mann–Whitney U test. Group means were AI = 4.387 and CI = 6.523. (**b**) Bar graph of Lactoferrin * *p*-value < 0.05 by T-Student test. Group means were AI = 247.268 and CI = 67.406. (**c**) Bar graph of Calprotectin * *p*-value < 0.05 by Mann–Whitney U test. Group means were AI = 162.148 and CI = 48.482. AI: allergic infants; CI: control infants.

ELISA analysis showed clear differences in the concentrations of the three fecal proteins between AI and CI. In the analysis of α1-antitrypsin, protein concentrations were higher in CI than in AI (*p*-value = 0.0068). As for lactoferrin, concentrations were higher in the AI compared to the CI (*p*-value = 0.0296). Finally, calprotectin concentrations followed a similar trend to lactoferrin, being higher in AI compared to CI. In the univariate statistical analysis, a significant *p*-value of 0.0132 was obtained. Figure 3 shows the bar graphs of these three fecal biomarkers between the two study groups.

### 2.3. Correlation Analysis between Fecal Proteins and Microbiota Functionality

The exploratory correlation analysis, performed with the Metaboanalyst 5.0 platform, between the three fecal proteins and all metabolic pathways obtained in the metagenomic sequencing yielded 39 routes. Notably, these routes included glycerophospholipid metabolism, the most significant pathway in the metagenomic analysis (Table 1). Pathways that were not related to immunity, inflammation, and gut microbiota were discarded from further analysis. After filtering, a total of 22 metabolic pathways were selected.

**Figure 4:**
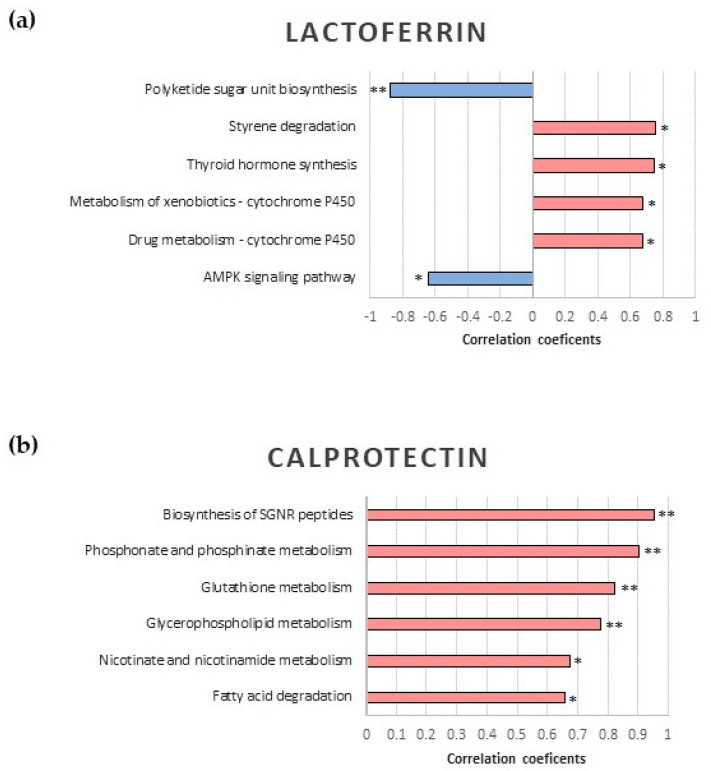
(**a**) Correlation plot for Lactoferrin; (**b**) correlation plot for Calprotectin; SGNR: siderophore group non-ribosomal; AMPK: AMP-activated protein kinase; * *p*-value < 0.05; ** *p*-value < 0.01.

It was observed that α1-antitrypsin had no significant correlations in the chosen pathways. On the other hand, in the correlation analysis between lactoferrin concentrations and the 22 metabolic pathways, six significant pathways were obtained. Finally, the correlation analysis of fecal calprotectin yielded six significant metabolic pathways, four of which had a *p*-value < 0.005. These results are shown in Figure 4.

## 3. Discussion

In this study, infants were recruited to investigate the effect of the microbiota on allergy development from a novel point of view involving fecal protein immunodetection and shotgun metagenomics.

The metagenomic analysis allowed the identification of 15 metabolic microbiome-encoded pathways associated with disease status, among which one of them stood out for its significant adjusted *p*-value and its possible relationship with CMA: the metabolism of glycerophospholipids. Changes in lipid metabolism at the mucosal level can lead to loss of epithelial barrier integrity and dysbiosis [45]. These changes and the immune responses that take place in the mucous membranes are related to a specific and systemic release of metabolites, with a corresponding alteration of their metabolic pathways [46,47]. In this study, four metabolic pathways related to lipid metabolism are increased in the AI group compared to CI. These data may suggest a potential disruption of intestinal barrier associated with the onset of food allergy.

On the other hand, clear differences were observed between the AI and CI groups in fecal proteins obtained by immunodetection. First, α1-antitrypsin was found to be significantly decreased in AI. Majamaa et al. [48] observed that an increased concentration of fecal α1-antitrypsin is associated with CMA in infants with atopic eczema. However, other authors analyzed α 1-antitrypsin levels along with a panel of different markers in patients with GIT disorders and found no significant differences for this protein [32,49]. In relation to CMA, there are scarce data, and the results are contradictory.

In this study, lactoferrin and calprotectin were found to be significantly elevated in the AI group, which is in line with previous literature. Lactoferrin is an abundant component of neutrophil-specific granules and can be released into serum upon neutrophil degranulation [40]. Increased fecal lactoferrin levels have been linked to diseases characterized by chronic inflammation of the GIT [40,50,51]. Borkowska et al. [50] described children diagnosed with Crohn’s disease or moderate ulcerative colitis as having significantly higher fecal lactoferrin concentrations than those with a mild or inactive disease. It has been described that lactoferrin increases the permeability of the bacterial cell membrane, resulting in the discharge of bacterial lipopolysaccharide and other cell contents from the outer membrane [38] that may lead to tissue inflammation. On the other hand, calprotectin measurement has been evaluated as a non-invasive marker of gastrointestinal inflammation, as its concentrations are known to correlate with the level of inflammation of the intestinal mucosa [52,53]. Based on this fact, it has been used for tracking intestinal conditions such as inflammatory bowel disease [27,54]. In relation to CMA, a notable example is the study of Qiu et al. [30], in which they noticed that the level of fecal calprotectin in infants with CMA decreased after dietary intervention, so it seems to be a promising biological indicator for controlling gastro-intestinal allergic diseases. Likewise, Beşer et al. [55] observed elevated calprotectin levels in children with CMA, suggesting its use as a biomarker. However, the evidence is conflicting, as other authors found no significant differences [31,56]. Both lactoferrin and calprotectin are released by the gastrointestinal tract in response to infection and mucosal inflammation and numerous studies have analyzed both [28,29,57,58,59]. In general, fecal markers have been found to be more accurate than serum markers. In this way, calprotectin and lactoferrin can differentiate inflammatory disease from functional bowel disorders. An example of this is a study conducted with patients with *Clostridium difficile* infection, where it was observed that both fecal calprotectin and lactoferrin were higher in those infected, especially in those with detectable toxin in the feces [59]. There are several studies pointing to lactoferrin and calprotectin, together with C-reactive protein, as reliable biomarkers of active inflammation in intestinal bowel disease (IBD) [29,51]. Therefore, in our study, we suggest that the increase of both proteins in the feces of AI compared to CI points to damage in the mucosa and intestinal inflammation produced by CMA.

In the correlation analysis, no significant metabolic pathways correlating with α1-antitrypsin were identified, but several pathways were identified to be associated with lactoferrin and calprotectin. Likewise, lactoferrin was inversely correlated with the biosynthesis of the polyketide sugar unit. Polyketides are secondary metabolites of bacteria, and it has been described that saturated fatty acids can be considered the smallest end product of the polyketide pathway. Moreover, the bacteriostatic activity of lactoferrin lead to the leakage of bacterial cell membrane components, such as fatty acids. Thus, our results suggest that lactoferrin may be related to a fatty acid increase during allergy development. These findings provide new insight into the physiological role of lactoferrin for the maintenance of cellular and tissue homeostasis [60].

Fecal calprotectin had a significant positive correlation with several metabolic pathways. The first correlation observed was with the fatty acid degradation pathway. Calprotectin has been described as having an essential role in inflammation-related processes, such as regulating the accumulation of neutrophils and macrophages, cytokine production, and fatty acid transport, among others. It can bind to unsaturated fatty acids, including arachidonic acid, and oleic acid [61], leading to structural changes that could be beneficial for their participation in the inflammatory process. On the other hand, short-chain fatty acids (SCFAs) are metabolites related to the gut microbiota, as they are the main source of energy for colonocytes [62]. It has also been reported that SCFAs can promote the differentiation of colon Tregs [63]. Increased fatty acid degradation could reduce the abundance of SCFAs, leading to a more pro-inflammatory environment with a lower number of Tregs and an increase in the inflammation marker calprotectin. Finally, in previous studies, gut microbiota, permeability, SCFAs, and inflammation have been shown to be altered in allergic diseases [63]. The correlation between fecal calprotectin and fatty acid degradation has also been studied in other diseases such as chronic stress [64] and Parkinson’s disease [65]. A possible alteration of fatty acid metabolism caused by CMA might be related to increased calprotectin levels in the AI group. Another metabolic pathway with a positive correlation with calprotectin was that of glutathione metabolism. Food allergy is associated with dysfunction of the intestinal epithelial barrier, which can be affected by oxidative stress. Some studies suggest that catalase and glutathione peroxidase concentrations correlate significantly with fecal calprotectin levels in patients with inflammatory bowel disease [66,67]. Interestingly, calprotectin levels have been also positively correlated with platelet aggregation and activation [68]. Although we have not analyzed in this manuscript platelet aggregation and activation, these previously published result data may be of great interest since it has been described that patients with a severe respiratory allergic phenotype, with or without a food allergy, present an alteration of platelet functions such as aggregation, adhesion, activation, and release of granules compared to moderate allergic phenotypes and non-allergic subjects [69]. Thus, it will be interesting to study platelet aggregation and activation in the context of CMA in the future. Finally, the correlation of calprotectin with the metabolism of glycerophospholipids stands out, as this pathway has a significant adjusted *p*-value in the metagenomic analysis. Schwarz et al. [70] have confirmed significantly higher fecal calprotectin levels in patients with Crohn’s disease than in healthy subjects. Phospholipids and glycerophospholipids may play several roles in the chronic inflammation of the gastrointestinal tract that characterizes Crohn’s disease. For instance, they might function as signaling molecules that stimulate the production of inflammatory mediators and activate immune cells. There is evidence that the intestinal phospholipid and glycerophospholipid profile may be altered in Crohn’s disease, with some studies demonstrating lower levels of certain species [71,72]. These alterations in phospholipid composition could play a role in the development of inflammation and, consequently, in the increased calprotectin levels observed not only in Crohn’s disease activity but also in other inflammatory diseases such as CMA. Likewise, the increase of calprotectin has been associated with obesity, a disease in which there are frequently higher levels of lipids in children, and the potential usefulness of this biomarker in the follow-up of their metabolic complications has been suggested [73].

The strengths of this study are the rigorous determination of CMA in the hospitals and health centers involved, the rigorous collection of epidemiological data from participants, and the use of high-yield, culture-independent techniques for the identification of the microbiota and the pathways involved. As limitations, we found that diet and the allergic status of infants were confounded, since AI infants fed cow’s milk-based formula (exclusively or as a complement to breastfeeding), were shifted to other alternatives such as partially hydrolyzed formula, extensively hydrolyzed formula, or hydrolyzed soybean formula at the time of diagnosis of CMA [74,75]. On the other hand, fecal samples were collected only once so changes in the microbiota over time (e.g., before and after allergy development) were not studied.

In this study, we have established that AI and CI groups vary in terms of fecal protein levels and the abundance of specific bacterial encoded pathways. Our findings further indicate that the AI group has altered lipid and fatty acid metabolisms as well as higher levels of lactoferrin and calprotectin. These metabolic changes may result in alterations in bacterial membranes that might cause modifications in lipid metabolism at the mucosal level [76]. The higher levels of lactoferrin and calprotectin pinpoint to intestinal inflammation and barrier disruption brought on by CMA. To what extent bacterial glycerophospholipids participate in this process should be clarified in future studies.

## 4. Materials and Methods

### 4.1. Study Design and Population

A longitudinal and observational case-control study approved by the Regional Committee on Ethics in Clinical Research of the Hospital Universitario Infantil Niño Jesús in Madrid was conducted in accordance with the ethical guidelines outlined in the Declaration of Helsinki and its amendments [77]. Infants were recruited into the Allergy Service of Hospital Universitario Infantil Niño Jesús, Hospital General Universitario Gregorio Marañón and five health centers in Madrid, Spain. All participants provided informed consent.

Fifty participants up to 8 months of age were recruited for this study: 34 infants with CMA (AI) and 18 healthy control infants (CI). Epidemiological data of the included infants are given in Table 2. Analysis of epidemiological data, including potential confounding factors such as age, diet, type of delivery, etc., was previously performed by our group [20]. Shotgun sequencing was performed on samples from infants aged 4–6 months, thus comprising 25 AI and 12 CI. This selection allowed us to standardize the AI and CI groups and to avoid biases due to the age of the infants and the introduction of solid foods. For fecal protein analysis, 20 samples from infants, 10 AI and 10 CI, were analyzed due to quantity limitations of the samples. Finally, correlation analysis was performed with paired data from 10 infants, 6 AI and 4 CI.

### 4.2. Inclusion and Exclusion Criteria

Infants up to 8 months with a clinical history of IgE-mediated food allergy and positive Skin Prick Test (SPT) to cow’s milk proteins (α-lactalbumin, β-lactoglobulin, and/or casein (Diater^®^, Madrid, Spain)) were included. Infants with a history consistent with CMA and a negative SPT were also included at their first visit if the test was positive after three months. All SPT were performed using standardized techniques in accordance with international guidelines [78].

The included CI had no symptoms related to CMA or any serious illness and were recruited from five health centers in Madrid.

Exclusion criteria were the intake of hydrolyzed formula for more than 2 weeks at diagnosis of CMA and antibiotic intake in the 3 months prior to study recruitment (also applicable to their mothers).

All participating centers applied the same recruitment protocols and questionnaires. Information was collected on personal data and risk factors for allergy in infants, such as age, sex, feeding regimen, mode of delivery, and use of antibiotics at birth for infants.

### 4.3. Sample Collection and Processing

Each participant received a sample collection kit designed and made by our group, which included: collection paper, a sterile stool collection container, gloves, a cold bag, an airtight bag, labels for coding and tracking the samples, and an easy-to-read guide, developed by our group, to facilitate sample collection in the private home [4]. Participants recorded the collection date of each sample and stored them in the home freezer at −20 °C for up to three days before taking them to the hospital. A total of 52 fecal samples (one per infant) were collected and stored at −80 °C before being processed for DNA and protein extraction.

### 4.4. Shotgun Metagenomics

#### 4.4.1. Sample Preparation

DNA was extracted from 200 mg of feces using the kit “QIAamp DNA Stool Mini Kit” (QIAGEN, Hilden, Germany) following the manufacturer’s instructions. To this protocol was added an additional step of membrane disruption using metal beads [79]. Negative controls were included in each extraction run. The extracted DNA was quantified using the Qubit^®^ 2.0 fluorometer following the protocol of the “dsDNA HS Assay” kit. Shotgun sequencing was performed on 29 samples from infants aged 4–6 months, that had a concentration greater than 0.2 μg/μL: 22 AI (AI_03, AI_04, AI_05, AI_08, AI_09, AI_11, AI_12, AI_13, AI_14, AI_16, AI_17, AI_19, AI_21, AI_22, AI_23, AI_24, AI_27, AI_29, AI_30, AI_31, AI_32, and AI_35) and 7 CI (CI_04, CI_05, CI_06, CI_09, CI_10, CI_11, and CI_12).

#### 4.4.2. Shotgun Sequencing

Metagenomic sequencing was performed using a NextSeq platform (Illumina, San Diego, USA) according to the manufacturer’s recommendations for obtaining paired-end sequences of 150 base pairs.

#### 4.4.3. Bioinformatics and Statistical Analysis

First, the paired-end Fastq files were filtered by quality with the Fastp application (version 0.20.1) [80]: low-quality bases were trimmed from the tail of each read, low complex reads were removed, and reads shorter than 35 bases were discarded. Next, all the reads passing the previous filters were mapped onto the *Homo sapiens* genome (GCA_000001405.28 GRCh38.p13 assembly) by means of Bowtie2 (version 2.4.2) [81]. Finally, the reads that did not align concordantly against the *Homo sapiens* genome were analyzed with the SqueezeMeta pipeline with co-assembly mode (version 1.3.1; database built on September 2020) [82]. Tabular outputs were generated from the SqueezeMeta results using the sqm2tables.py SqueezeMeta script, ignoring unclassified reads in the TPM calculation.

To identify significant differences in abundance between KEGG modules, metabolic pathways and KOs between AI and CI groups, the DESeq2 v.1.24.0 algorithm was used with default parameters in R software R4.0.0 [83]. False Discovery Rate (FDR) was applied to adjust for multiple hypothesis tests using the “doTestDESeq2” test. In addition, to study differences in the overall microbiome composition, permutational multivariate analysis of variance using Bray–Curtis distance matrices (ADONIS) [84] was performed. Furthermore, the script doAdonisFromDistances.R was used to perform a PCoA and a Permanova with an associated *p*-value. Metabolic pathways with an adjusted *p* value < 0.05 per FDR were considered significant.

### 4.5. Fecal Protein Immunodetection (ELISA)

A total of 20 infant feces samples of sufficient quantity, 10 AI (AI_16, AI_20, AI_22, AI_26, AI_28, AI_29, AI_31, AI_32, AI_34, and AI_35) and 10 CI (CI_01, CI_03, CI_04, CI_05, CI_06, CI_07, CI_11, CI_16, CI_23, and CI_24), were analyzed by ELISA for the concentrations of three proteins: α1-antitrypsin, lactoferrin, and calprotectin.

#### 4.5.1. Sample Preparation

The 20 selected feces samples were homogenised, and 20 aliquots of 180 mg were prepared. Next, protein extraction was performed by a 1:9 dilution (sample: PBS) in three serial dilutions. First, 540 μL of PBS were added to the Eppendorf tubes with 180 mg of stool sample and a 5-min sonication was performed. The samples were then centrifuged (10,000 rpm, 10 min, 4 °C) and the supernatants were transferred to another Eppendorf tube. On the remaining pellet, the extraction process was repeated twice. The pool of supernatants obtained was divided into 3 aliquots of 400 μL for the different protein analyses.

#### 4.5.2. ELISA Protocol

The appropriate dilutions for each protein were calculated and prepared, considering the expected concentrations obtained in the literature search. The final dilutions were 1:250, 1:250, and 1:100 for α1-antitrypsin, lactoferrin, and calprotectin, respectively. Once the corresponding dilutions were made in all the samples, the protocol of the commercial kit for Fine Test^®^ ELISA (Wuhan, China) was followed for each of the study proteins.

The optical density of the results was analyzed by a Varioskan plate reader at 450 nm and 620 nm in two measurements. With the data obtained from the 7 standard patterns, a standard curve of 5 parameters was obtained that was used to interpolate the sample data.

#### 4.5.3. Data Processing and Statistical Analysis

For data processing, the absorbances measured at 450 nm minus the measurements at 620 nm in each sample were subtracted and the duplicates of the first measurement were averaged. Then, the same process was conducted with the second measurement and an average of the measurements obtained in each sample was made.

Statistical analysis was performed with GraphPad Prism v8.0.1 software (GraphPad Software). This program was used to calculate the concentrations with the calibration curve, the statistical value with parametric and non-parametric analysis and the bar graph of each study protein. Comparisons between experimental groups were carried out in 3 steps. First, a nonlinear fit analysis and subsequent interpolation of concentrations were performed. Then, we checked if within each group the data fitted the normal distribution, using the Shapiro–Wilk normality test. Finally, parametric analyses were performed for unpaired samples (T-Student). When groups did not behave following a normal distribution, the nonparametric Mann–Whitney U test was applied, which compares the means of the experimental groups. In all analyses, a *p*-value < 0.05 was considered significant.

### 4.6. Correlation Analysis

The correlation study between the results obtained by metagenomics and the ELISA technique was performed with a total population of 10 infants, 6 AI (AI_16, AI_22, AI_29, AI_31, AI_32, and AI_35) and 4 CI (CI_04, CI_05, CI_06 and CI_11). The sample size was reduced due to two factors: the amount of sample collected in many cases was not sufficient to carry out both analysis and not all infants were included in the study before sequencing, so data were obtained from the ELISA analysis but not from metagenomics in some of them.

Initially, the important biological pathways were manually screened. The information for each protein was then combined in a comma-separated values (CSV) file with the information for the chosen metabolic pathways in accordance with the specifications of the Metaboanalyst platform. For the statistical correlation analysis, the data were entered into the Metaboanalyst 5.0 (https://www.metaboanalyst.ca, accessed on 29 June 2021) online platform with auto-scaling and without normalization nor transformation. The metabolic pathway correlation by pairs and their grouping using Pearson’s correlation coefficient were displayed on the research proteins’ heatmap. The *p*-values of the connection with each of the metabolic pathways were then determined by doing a pattern study for each protein.

## 5. Conclusions

In this work, we have confirmed that there are differences, both in metagenomic pathways and in fecal protein levels, between AI and CI groups. Our results show an alteration in lipid and fatty acid metabolisms, as well as an increase in lactoferrin and calprotectin in the AI group. These findings could point to intestinal inflammation and disturbance possibly brought on by food allergy. Previous work by our group has shown that epithelial barrier integrity is compromised in severe allergic phenotypes, regardless of the triggering allergen, and that this has systemic consequences [85,86]. In our study, the integrity of the epithelial barrier of allergic infants could be compromised beforehand due to metabolic alterations that help establish the allergic phenotype. Dysbiosis could therefore be a consequence of the disease. However, prospective studies in this regard would be necessary to confirm this hypothesis.

On the other hand, since allergy and diet factors were confounded in AI and CI, prospective studies that include different time intervals during the first months of life of infants are necessary to understand the role of differences at the level of intestinal microbiota between AI and CI in the development of allergy. Finally, the identification by omics techniques of the altered metabolic pathways and metabolites involved, such as lipid mediators, would complement our findings and help discover additional mechanisms by which the intestinal microenvironment of the first years of life influences the risk of developing a food allergy.

## Figures and Tables

**Table 1:** Significant metabolic pathways (*p*-value < 0.05) between AI and CI. KP: KEGG pathway; FC: fold change.

Metabolic Pathway	KP	*p*-Value	Adj. *p*-Value	log2FC
Glycerophospholipid metabolism *	ko00564	0.000008	0.002419	0.243
Nicotinate and nicotinamide metabolism	ko00760	0.001577	0.246808	0.233
Primary immunodeficiency	ko05340	0.005161	0.538457	0.328
Inositol phosphate metabolism	ko00562	0.008359	0.654097	0.451
Glycerolipid metabolism	ko00561	0.016640	0.892611	0.183
Phosphatidylinositol signaling system	ko04070	0.017552	0.892611	0.203
Lipoic acid metabolism	ko00785	0.022028	0.892611	0.328
Atrazine degradation	ko00791	0.028442	0.892611	0.983
Glycine, serine, and threonine metabolism	ko00260	0.028572	0.892611	0.139
Chlorocyclohexane and chlorobenzene degradation	ko00361	0.030698	0.892611	0.404
Biosynthesis of unsaturated fatty acids	ko01040	0.035266	0.892611	0.337
Carbon fixation pathways in prokaryotes	ko00720	0.035961	0.892611	−0.175
Ubiquitin system	ko04121	0.046358	0.892611	0.461
Limonene and pinene degradation	ko00903	0.047001	0.892611	0.438

* Pathway with an adjusted *p*-value < 0.05.

**Table 2:** Main demographic and clinical characteristics of the infants included in the study. AI: allergic infant; CI, control infant; M: male; F: female; BM, breast milk; MF: milk formula; H: hydrolyzed formula; -: missing information.

Sample ID	Age (Months)	Gender	Condition	Delivery Mode	Antibiotics in Labor	Feeding
AI_01	4	M	Allergic	C-section	Yes	BM+H
AI_02	5	F	Allergic	C-section	No	BM
AI_03	5	M	Allergic	Vaginal	No	BM+H
AI_04	4	M	Allergic	C-section	No	BM+H
AI_05	5	F	Allergic	Vaginal	Yes	BM+H
AI_06	3	F	Allergic	Vaginal	No	BM+H
AI_07	2	M	Allergic	Vaginal	No	H
AI_08	6	F	Allergic	Vaginal	No	H
AI_09	6	M	Allergic	Vaginal	No	H
AI_10	3	F	Allergic	C-section	Yes	BM+MF
AI_11	5	M	Allergic	Vaginal	No	H
AI_12	6	F	Allergic	Vaginal	No	BM
AI_13	5	M	Allergic	Vaginal	No	H
AI_14	5	F	Allergic	Vaginal	No	BM+MF
AI_15	3	M	Allergic	Vaginal	No	BM+H
AI_16	4	F	Allergic	Vaginal	Yes	BM
AI_17	5	F	Allergic	Vaginal	No	H
AI_19	5	F	Allergic	Vaginal	No	BM+H
AI_20	7	M	Allergic	Vaginal	No	BM
AI_21	6	F	Allergic	Vaginal	No	BM+MF
AI_22	6	F	Allergic	C-section	No	BM
AI_23	6	F	Allergic	Vaginal	No	H
AI_24	6	F	Allergic	Vaginal	No	BM+H
AI_26	8	F	Allergic	Vaginal	No	BM+H
AI_27	5	M	Allergic	Vaginal	No	BM+H
AI_28	7	M	Allergic	Vaginal	Yes	BM
AI_29	5	F	Allergic	Vaginal	Yes	BM+H
AI_30	6	M	Allergic	Vaginal	No	H
AI_31	4	F	Allergic	Vaginal	No	H
AI_32	6	M	Allergic	C-section	No	H
AI_33	4	F	Allergic	Vaginal	No	BM+H
AI_34	3	M	Allergic	Vaginal	No	BM+H
AI_35	6	M	Allergic	Vaginal	No	H
AI_36	1	F	Allergic	Vaginal	No	H
CI_01	6	M	Non-allergic	Vaginal	No	BM
CI_02	7	M	Non-allergic	Vaginal	No	BM+MF
CI_03	6	F	Non-allergic	Vaginal	No	BM+MF
CI_04	6	F	Non-allergic	Vaginal	Yes	BM+MF
CI_05	6	M	Non-allergic	Vaginal	No	MF
CI_06	6	M	Non-allergic	Vaginal	No	MF
CI_07	8	M	Non-allergic	Vaginal	No	BM+MF
CI_08	5	F	Non-allergic	Vaginal	No	MF
CI_09	5	F	Non-allergic	C-section	No	MF
CI_10	5	F	Non-allergic	C-section	No	MF
CI_11	5	F	Non-allergic	Vaginal	No	BM
CI_12	4	M	Non-allergic	Vaginal	No	MF
CI_13	3	F	Non-allergic	Vaginal	No	MF
CI_14	4	F	Non-allergic	Vaginal	No	BM+MF
CI_15	1	F	Non-allergic	Vaginal	No	BM+MF
CI_16	3	F	Non-allergic	C-section	Yes	BM+MF
CI_23	4	F	Non-allergic	C-section	-	BM+MF
CI_24	3	F	Non-allergic	C-section	No	BM+MF

## Data Availability

The shotgun metagenomics sequencing files and metadata have been deposited in the Sequence Read Archive (SRA) at NIH and are available under accession number SUB13444105.
